# Influence of pH Modification on Catalytic Activities of Metal-Doped IrO_2_ Nanoparticles

**DOI:** 10.1038/s41598-019-42358-9

**Published:** 2019-04-09

**Authors:** Joo Yeon Kim, Hangil Lee

**Affiliations:** Department of Chemistry, Sookmyung Women’s University, Seoul, 04310 Republic of Korea

## Abstract

The effects of pH variation on the catalytic activity of IrO_2_ nanoparticles (NPs) doped with Cr (an early transition metal) or Ni (a late transition metal) depending on the amount of defect structures on the NP surfaces were analyzed. It was found that both Cr@IrO_2_ and Ni@IrO_2_ NPs, fabricated under basic conditions (pH = 13.5) denoted as Cr@IrO_2_-B and Ni@IrO_2_-B, respectively, were the best catalysts among the eight tested ones. Moreover, it was confirmed that variation in pH resulted in the changes in the surface area (defect structure), which were considered to be responsible for the changes in the catalytic properties of these NPs. For the oxygen evolution reaction, these NPs exhibited relatively smaller overpotential (η) values than other tested Cr@IrO_2_- and Ni@IrO_2_-containing NPs. Furthermore, methylene blue degradation analysis and OH radical formation experiments by benzoic acid showed the same trend. Thus, we confirmed that the catalytic activity of transition metals doped IrO_2_ NPs fabricated under basic conditions can be improved.

## Introduction

Metal oxide nanoparticles (MO NPs) have various advantages such as low cost, high stability, and better catalytic properties than those of metals, and are thus being used as efficient catalysts since several decades^1–9^. MO NPs such as those of TiO_2_, SnO_2_, and CeO_2_ are particularly stable catalysts and have hence been synthesized in various forms (sizes, shapes, etc.)^10^. Since MO NPs are also used in batteries, many researchers are still studying them particularly to enhance their effectiveness^11–13^. Iridium oxide nanoparticles (IrO_2_ NPs) also constitute smart materials having impressive applications as catalysts^14–17^, components in fuel cells^18–20^, and others^21–23^. They have even attracted the attention as catalysts for the oxygen evolution reactions (OER) in batteries^24–27^.

Meanwhile, several studies have identified the catalytic efficiency of MO NPs to be closely correlated with the concentration of defect structures formed on their surface, and in particular, dependent on their surface area^28–32^. In other words, the defect structure on the IrO_2_ NPs has been proposed as the active sites of electrochemical oxidation and photocatalytic reactions through the excitation from the O-*p* band to the Ir-*d* band^33^. Hence, many research groups have been tried via inserting dopants such as transition metals into these NPs to enhance their catalytic activities, because the doped transition metals can increase the defect structure by the charge transfer through reaction with the IrO_2_ NPs^34,35^. In case of IrO_2_ NPs, transition metal dopants can access various oxidation states and then consequentially change their electronic states of IrO_2_ NPs. Although the active sites of the IrO_2_ NPs can be different depending on the catalytic reactions due to the different chemicals and reaction pathways, similar strategies such as doping have been successfully demonstrated to enhance the catalytic activity of IrO_2_ NPs.

Various other studies showed that although undoped IrO_2_ NPs, as well as those doped with metallic Cr (an early transition metal) or Ni (a late transition metal), display relatively poor catalytic properties, a modification of their surface affects their catalytic activities. These studies also showed that the changes in pH levels of the environment of NPs may modify their activities^36–38^.

In the current work, we synthesized recently well-studied IrO_2_ NPs doped with Cr and Ni, denoted as Cr@IrO_2_ and Ni@IrO_2_ NPs, analyzed the effect of pH variation on their catalytic properties, and then systematically compared their catalytic activities with those of NPs which pH levels were not adjusted. We characterized the electronic structures and morphologies of Cr@IrO_2_ and Ni@IrO_2_ NPs by using high-resolution photoemission spectroscopy (HRPES) and scanning electron microscopy (SEM), respectively. We specifically aimed to compare the catalytic properties of these NPs and to determine the driving force of each catalyst, with the ultimate purpose of deriving the pH condition that would yield the highest catalytic efficiency for eight tested NPs. To figure out NPs that provide the best catalytic efficiency, we tested their catalytic activity with the change of overpotential by OERs, methylene blue (MB) degradation, and OH radical formation of benzoic acid in aqueous solutions.

## Results and Discussion

### Characterization of electronic structure

**Figure 1:**
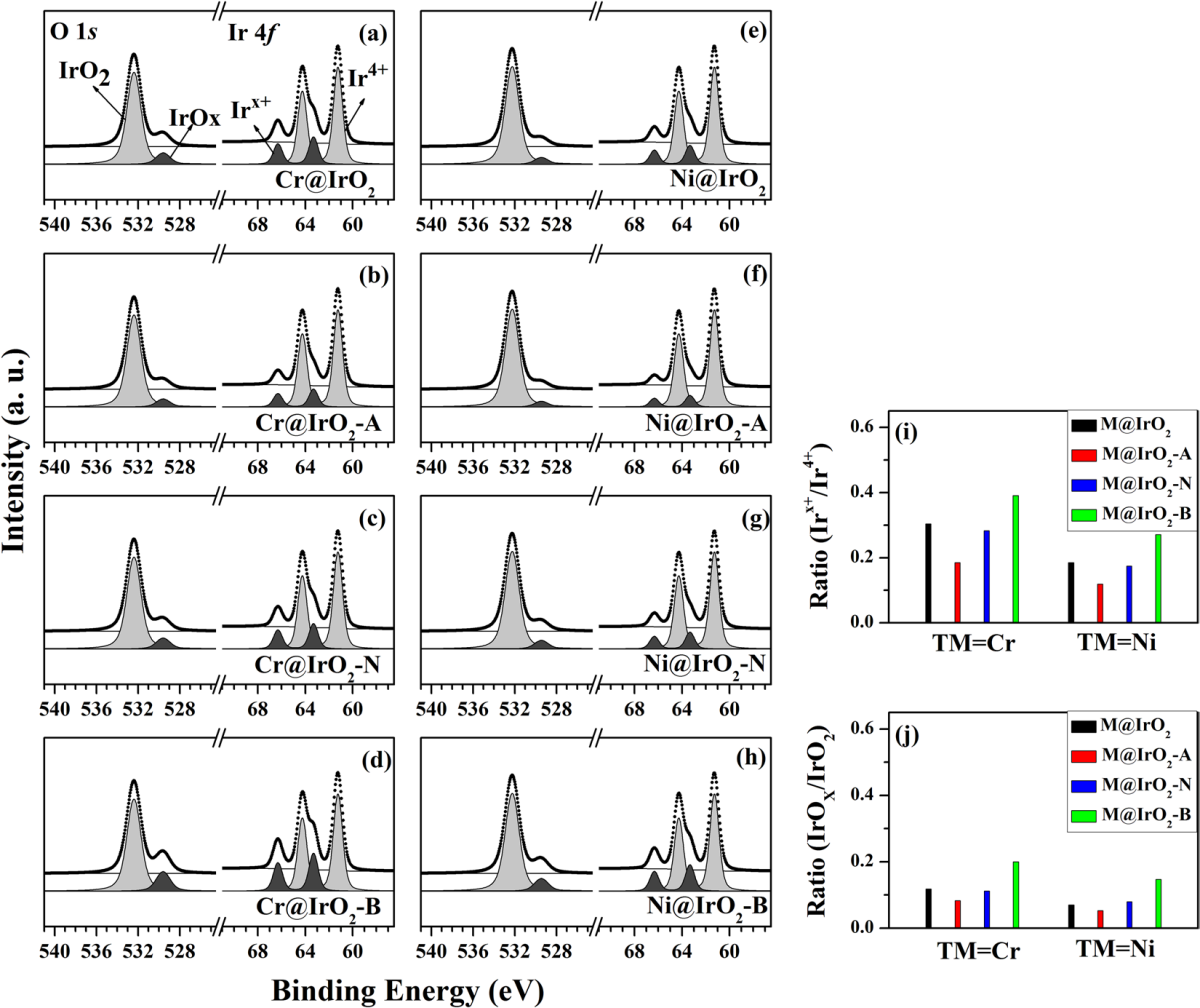
HRPES results for (**a**) Cr@IrO_2_ NPs, (**b**) Cr@IrO_2_-A NPs, (**c**) Cr@IrO_2_-N NPs, and (**d**) Cr@IrO_2_-B NPs, as well as for (**e**) Ni@IrO_2_ NPs, (**f**) Ni@IrO_2_-A NPs, (**g**) Ni@IrO_2_-N NPs, and (**h**) Ni@IrO_2_-B NPs. TM@IrO_2_-A, TM@ IrO_2_-N, and TM@IrO_2_-B NPs indicate transition metal (TM = Cr or Ni)-doped IrO_2_ NPs subjected to acid (pH = 1.5), neutral (pH = 7.0), and base (pH = 13.5) treatments, respectively. (**i**) The intensity of the Ir^x+^ peak divided by that of the Ir^4+^ peak for each of the eight samples. (**j**) The intensity of the IrO_x_ peak divided by that of the IrO_2_ peak for each of the eight samples.

Core-level spectra (Ir 4*f* and O 1*s*) of the eight IrO_2_-containing samples (TM@IrO_2_, TM@IrO_2_-A, TM@IrO_2_-N, and TM@IrO_2_-B NPs; TM = Cr or Ni) were obtained by HRPES (Fig. 1(a–h)) to analyze the differences in their electronic properties. As shown in the figures, all the spectra contained two distinctive features: peaks corresponding to Ir 4*f*_7/2_ peaks at 61.3 eV (Ir^4+^) and 63.4 eV (Ir^x+^; defect structure induced peak), and O 1*s* peaks at 532.4 eV (IrO_2_) and 529.6 eV (IrO_x_; defect structure induced peak). When comparing the spectra of different samples, we focused on the variation in the intensities of the peaks induced by the defect structures (i.e., the Ir^x+^ and IrO_x_ peaks). The intensities of these peaks obtained from both TM@IrO_2_-B NPs (TM = Cr or Ni; treatments under basic conditions) were observed to be larger than those of other samples (Fig. 1(i,j)). The Ir^x+^ and IrO_x_ species are related to the defect structures in IrO_2_ NPs resulting from charge compensation^39–41^. The oxygen atoms removed from IrO_2_ NPs have been proposed to either form Cr or Ni oxide, or remain as oxygen vacancies. Table 1 shows the intensity ratio of the defect structure peak divided by the intensity of the defect-induced peak and the metal oxide-induced peak for each of the eight spectra shown in Fig. 1.

**Table 1:** The intensity of the defect structure peak divided by the intensity of the defect and metal peak for each of the eight samples.

	Intensity comparison	Untreated	Acid (pH = 1.5)	Neutral (pH = 7.0)	Base (pH = 13.5)
Cr@IrO_2_ NPs	${I}_{{\rm{Ir}}}^{{\rm{x}}+}/{I}_{{\rm{Ir}}}^{4+}$	0.304 ± 0.015	0.185 ± 0.009	0.283 ± 0.014	**0**.**391** ± 0.019
	*I*_IrOx_/*I*_IrO2_	0.118 ± 0.006	0.083 ± 0.004	0.112 ± 0.006	**0**.**213** ± 0.010
Ni@IrO_2_ NPs	${I}_{{\rm{Ir}}}^{{\rm{x}}+}/{I}_{{\rm{Ir}}}^{4+}$	0.185 ± 0.009	0.119 ± 0.006	0.174 ± 0.009	**0**.**271** ± 0.013
	*I*_IrOx_/*I*_IrO2_	0.071 ± 0.004	0.053 ± 0.003	0.082 ± 0.004	**0**.**147** ± 0.007

### Characterization of catalytic activities

**Figure 2:**
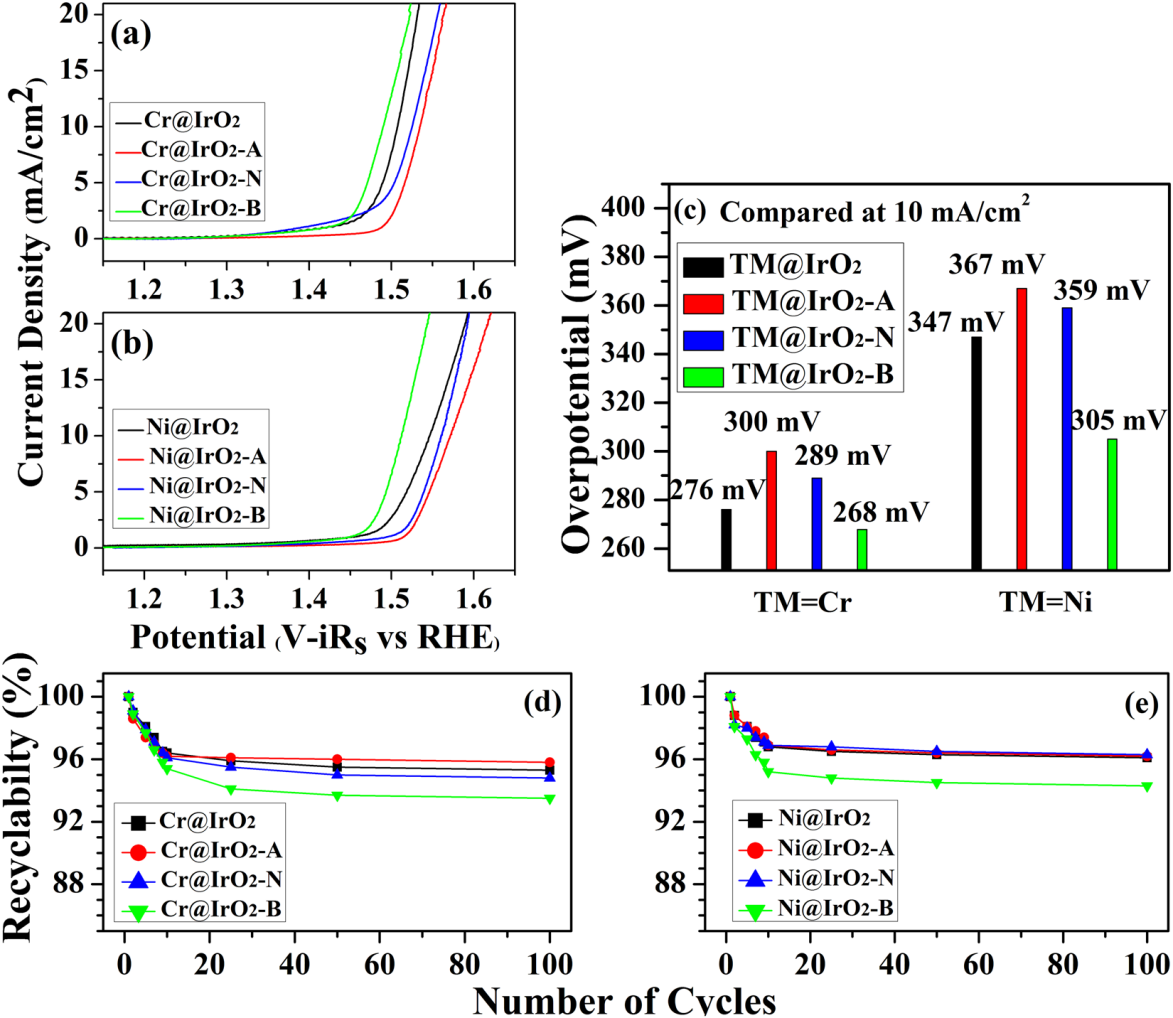
Current density-potential curves with iR_s_ correction for (**a**) Cr@IrO_2_ NPs, Cr@IrO_2_-A NPs, Cr@IrO_2_-N NPs, and Cr@IrO_2_-B NPs, (**b**) Ni@IrO_2_ NPs, Ni@IrO_2_-A NPs, Ni@IrO_2_-N NPs, and Ni@IrO_2_-B NPs measured in 1 M KOH, and (**c**) the plots of their overpotential obtained at a current density of 10 mA/cm^2^. Recyclability for up to 100 cycles of measurements of (**d**) Cr@IrO_2_ NPs, Cr@IrO_2_-A NPs, Cr@IrO_2_-N NPs, and Cr@IrO_2_-B NPs, and (**e**) Ni@IrO_2_ NPs, Ni@IrO_2_-A NPs, Ni@IrO_2_-N NPs, and Ni@IrO_2_-B NPs.

In order to compare the electrocatalytic activities of the investigated samples, we recorded the electrochemistry measurements^42–44^. The electrocatalytic OER reactions of TM@IrO_2_ NPs (TM = Cr or Ni) were evaluated in the alkaline 1 M KOH electrolyte condition (Fig. 2(a,b)). As shown in the figures, Cr@IrO_2_-B and Ni@IrO_2_-B NPs (green colored plots) displayed greater OER activities than did the other Cr- and Ni@IrO_2_-containing NPs, with the overpotential (η) values of 258 mV and 305 mV, respectively (Fig. 2(c)). It is notable that the highest OER activities were achieved for those Cr- or Ni@IrO_2_ NPs that also, according to the HRPES results above, showed the most defect-related features. Therefore, we propose that the defects in TM@IrO_2_ NPs are the active sites that result in the higher electrochemical OER activities (lower overpotential values).

In addition, the ability of eight tested NPs to maintain an efficient catalytic activity in the oxidation reaction for many cycles is an important factor that determines their practical applicability. To test this, the oxidation reaction for each sample was run for 100 cycles, and the results are shown in Fig. 2(d,e). All the samples were found to be relatively stable as measured by their abilities to catalyse the oxidation reaction. More specifically, in the first ten cycles, there was a slight decrease in the adlayer desorption resulting in a 4% decrease compared to the first cycle, but there was no further significant decrease in subsequent 90 cycles. As a result, we considered all eight samples to stably catalyse the oxidation reaction, albeit with some differences between their values. Even the Cr@IrO_2_-B and Ni@IrO_2_-B samples showed only a modest decrease, to 93.5 and 94.3%, respectively, of their initial values. Thus, all synthesized NPs can be considered to be relatively stable catalysts. The modest decrease observed for the Cr@IrO_2_-B and Ni@IrO_2_-B samples may be due to some sample desorption or deformation that possibly occurred during the oxidation reaction. Our results showed that we were able to improve (i.e., decrease) the overpotential of TM@IrO_2_ NPs, especially Ni@IrO_2_ NPs, by appropriately changing pH of these NPs, and suggested a general way for improving the catalytic efficiency of such NPs.

**Figure 3:**
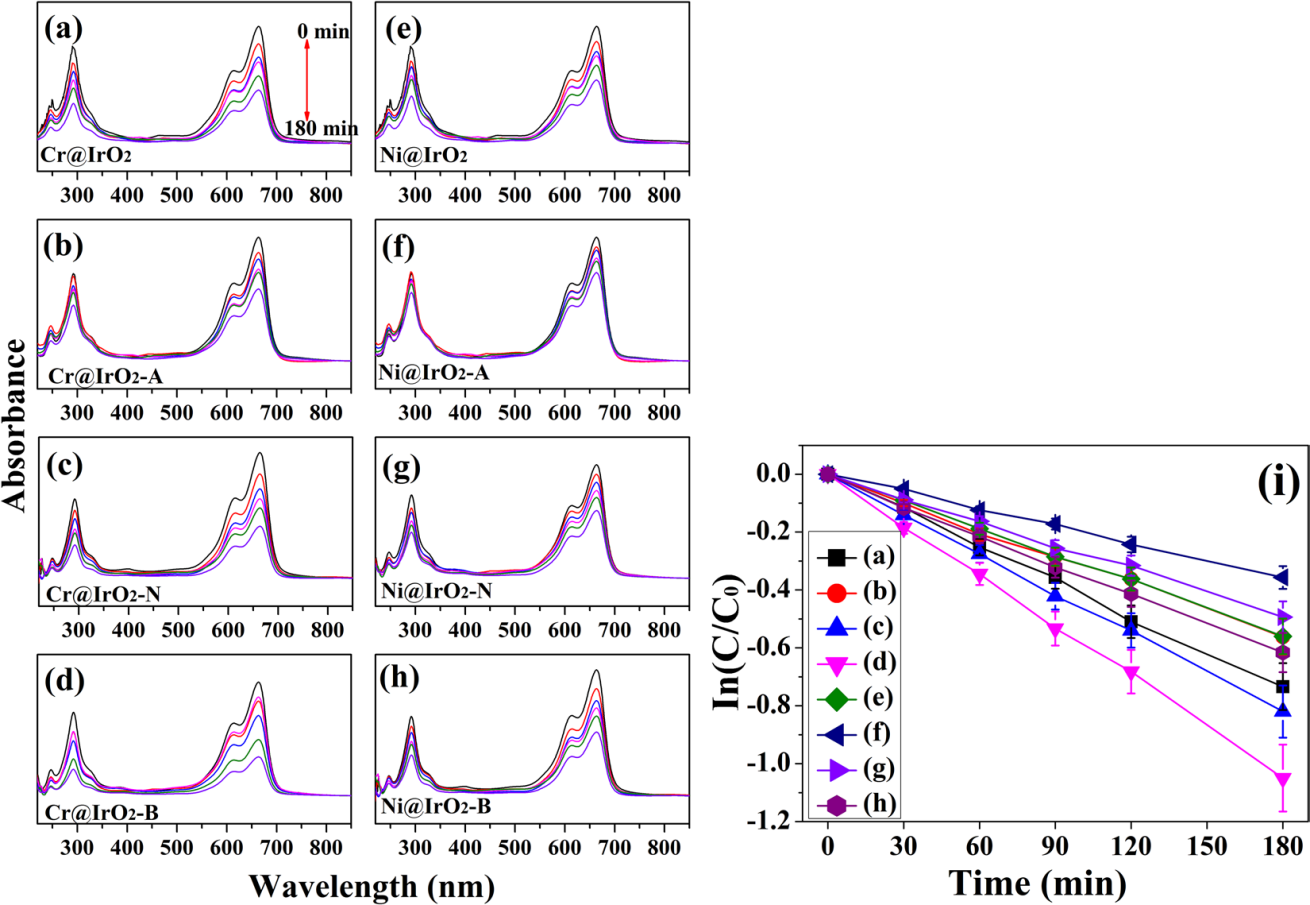
Degradation of methylene blue (0.15 mM) with (**a**) Cr@IrO_2_ NPs, (**b**) Cr@IrO_2_-A NPs, (**c**) Cr@IrO_2_-N NPs, and (**d**) Cr@IrO_2_-B NPs, (**e**) Ni@IrO_2_ NPs, (**f**) Ni@IrO_2_-A NPs, (**g**) Ni@IrO_2_-N NPs, and (**h**) Ni@IrO_2_-B NPs. (**i**) The plot of MB intensity variations for the eight tested samples.

MB degradation analysis was carried out to compare the catalytic efficiencies of the as-prepared samples. As shown in Fig. 3(a–f), the samples (TM@IrO_2_, TM@IrO_2_-A, TM@IrO_2_-N, and TM@IrO_2_-B NPs; TM = Cr or Ni) present a negligible extent of dye degradation. While slow degradation occurred, approximately 30–60% of MB degraded in 180 minutes under the same conditions for all samples. As shown in the Fig. 3(a–d), the catalytic activity of Cr@IrO_2_-B is better than those of other samples, as expected; in case of Cr@IrO_2_-B NPs, MB degraded by nearly 35% in 180 minutes.

In addition, among the four differently treated Ni@IrO_2_ NPs, Ni@IrO_2_-B NPs (55%) shows the best catalytic activity, as expected (see Fig. 3(e–h)). These results, along with those of OER shown in Fig. 2, indicate that the catalytic properties of TM@IrO_2_ NPs can be improved by treating them under basic conditions. We could also confirm that this result is related to the surface defect structures (or surface areas) of NPs. Table 2 shows the MB degradation values at 30 min and 180 min for each of the eight spectra shown in Fig. 3.

**Table 2:** The intensity variation for MB degradation of the eight tested samples at 30 min and 180 min.

	Intensity C/C_0_	Untreated	Acid	Neutral	Base
Cr@IrO_2_ NPs	30 min	0.75 ± 0.068	0.82 ± 0.073	0.72 ± 0.065	**0**.**68** ± 0.061
	180 min	0.48 ± 0.043	0.57 ± 0.051	0.44 ± 0.039	**0**.**35** ± 0.032
Ni@IrO_2_ NPs	30 min	0.81 ± 0.073	0.89 ± 0.080	0.79 ± 0.071	**0**.**77** ± 0.069
	180 min	0.57 ± 0.051	0.70 ± 0.063	0.61 ± 0.055	**0**.**55** ± 0.049

As shown in the plot (Fig. 3(i)), the degradation rate of TM@IrO2 NPs (TM; Cr and Ni) by MB was observed to be a function of time. In general, as changes in foreign materials follow a linear function (first-order function), we used a well-known formula, and experimental data were fitted to the following Eq. (1):

1 $$\mathrm{ln}\,(C/{C}_{0})=-\,kt$$

where *C* and *C*_0_ are absorbance of the M at time *t* and at time 0. *k* is the rate constant (min^−1^) and *t* is the time (min.).

When ln(*C/C*_0_) was plotted against time, a linear relationship was obtained. As can be seen from the first-order reaction plot shown in Fig. 3(i), the rates of change of Cr@IrO_2_-B and Ni@IrO_2_-B NPs are remarkably higher than those of other NPs. This indicates that the photocatalytic activity of the two NPs is better than that of the other NPs. It can therefore be confirmed that the photocatalytic property of TM@IrO_2_ NPs can be enhanced under basic condition (pH = 13.5).

We also calculated the rate constant value (*k*) for the change in concentration (ln(*C*/*C*_0_)) with time (min.) for the eight tested samples. In detail, the apparent rate constant for the decay of the reactant for Cr@IrO_2_ NPs was 5.73 × 10^−3^ min^−1^ when using Cr@IrO_2_-B NPs, greater than the cases using other NPs (4.11 × 10^−3^ min^−1^ for Cr@IrO_2_, 3.06 × 10^−3^ min^−1^ for Cr@IrO_2_-A, 4.53 × 10^−3^ min^−1^ for Cr@IrO_2_-N). In case of Ni@IrO2 NPs, we can confirm the same trends; 3.13 × 10^−3^ min^−1^ for Ni@IrO_2_, 2.07 × 10^−3^ min^−1^ for Ni@IrO_2_-A, 2.67 × 10^−3^ min^−1^ for Ni@IrO_2_-N, and 3.33 × 10^−3^ min^−1^ for Ni@IrO_2_-B. In other word, the significantly higher rate constant for the degradation reaction in the presence of Cr@IrO_2_-B or Ni@IrO_2_-B NPs would be attributed to the higher surface area of the NPs compared with those of other NPs.

As mentioned above, the improvement of the catalytic properties of IrO_2_ NPs has previously been shown to be closely related to the concentration of defect structures (or increased surface area) in the samples. In addition, we found that the intensities of the HRPES defect-induced peaks, i.e., those corresponding to Ir^x+^ or IrOx defect-induced structures, differed for NPs with different pH treatments (Fig. 1), and were closely related to the OER (Fig. 2) and MB degradation results (Fig. 3).

### Defect structure analysis

**Figure 4:**
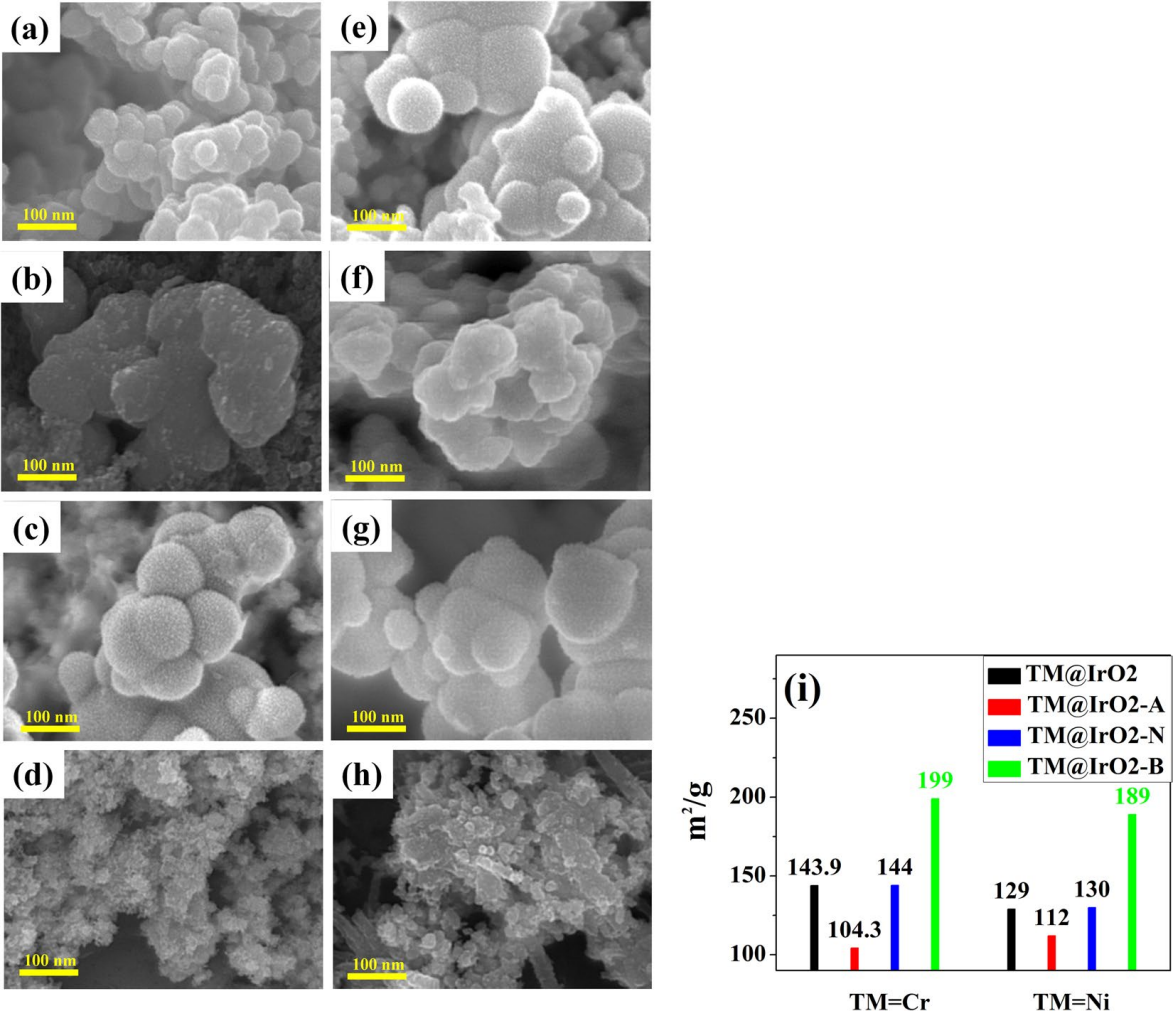
SEM images and BET measurements of (**a**) Cr@IrO_2_ NPs, (**b**) Cr@IrO_2_-A NPs, (**c**) Cr@IrO_2_-N NPs, (**d**) Cr@IrO_2_-B NPs, (**e**) Ni@IrO_2_ NPs, (**f**) Ni@IrO_2_-A NPs, (**g**) Ni@IrO_2_-N NPs, and (**h**) Ni@IrO_2_-B NPs. (**i**) BET measurements for the eight tested samples.

To provide a direct evidence and support the experimental results, we analyzed the SEM images (Fig. 4(a–h)) and BET measurements (Fig. 4(i)) of the samples. The SEM images of all eight samples showed protrusions on the surfaces of NPs. The protrusions on untreated NPs (Cr@IrO_2_ and Ni@IrO_2_; see Fig. 4(a,e)) were similar in size and shape to those on NPs treated under pH = 7 conditions (Cr@IrO_2_-N and Ni@IrO_2_-N; see Fig. 4(c,g)). Particularly interesting results were observed for the acid- and base-treated samples: the two acid-treated samples (Cr@IrO_2_-A and Ni@IrO_2_-A; see Fig. 4(b,f)) showed smooth protrusions, attributed to a low concentration of defect structures (or decreased surface area), while base-treated NPs (Cr@IrO_2_-B and Ni@IrO_2_-B; see Fig. 4(d,h)) showed rougher surfaces, with irregular flakes and very small protrusions, attributed to a high concentration of defect structures (or increased surface area). The BET experiments were performed with the hope of deriving a relationship between the specific surface areas of NPs and their catalytic properties. The average surface area of base-treated Cr@IrO_2_ NPs was determined to be significantly greater than those of untreated, pH 7-treated, and acid-treated Cr@IrO_2_ NPs. Similarly, the average surface area of base-treated Ni@IrO_2_ NPs was also significantly greater than those of other Ni@IrO_2_ NPs. Thus, the BET experiments revealed a significant effect of pH on the surface area of each sample.

As shown in Tables 1 and 2, a correlation was found between the results of the BET surface area analyses (Fig. 4(i)) and the concentration of defect structures indicated by the intensities of the corresponding HRPES peaks. By characterizing the electronic structures of eight types of TM@IrO_2_ NPs (TM = Cr or Ni), and the oxidation reactions catalyzed by them, we found that Cr@IrO_2_-B and Ni@IrO_2_-B NPs showed better OER (specifically, better electrochemical oxidation of water) and MB degradation performances than did the other Cr@IrO_2_- and Ni@IrO_2_-containing NPs (TM@IrO_2_, TM@IrO_2_-A, or TM@IrO_2_-N), respectively.

We made several sets of interpretations from our experimental results. The analysis of the HRPES results (Fig. 1) indicated a higher concentration of defect structures in base-treated NPs, (i.e., Cr@IrO_2_-B and Ni@IrO_2_-B) than in other six TM@IrO_2_ NPs (TM = Cr or Ni), and the analysis of the SEM and BET results (see Fig. 4) indicated the concentration of defect structures to be proportional to the surface area of NPs. Note in this regard that the concentration of defect structures has been shown in earlier studies to be positively associated with the quality of the catalyst^29,30^. Also, our results were consistent with the possibility that active radicals formed on the surface of IrO_2_ NPs, since the reaction of OH ions with the holes of IrO_2_ NPs has been shown to usually generate more ·OH radicals in basic conditions than in other conditions^45–47^.

To confirm that the OH radicals of eight tested NPs synthesized by their treatment under basic conditions (pH = 13.5) were generated in large amounts, the radical reaction of benzoic acid was performed.

2 $$\begin{array}{c}{{\rm{C}}}_{6}{{\rm{H}}}_{5}-{\rm{COOH}}+\cdot {\rm{OH}}\mathop{\longrightarrow }\limits^{hv}\,\mathrm{HO}-{{\rm{C}}}_{6}{{\rm{H}}}_{5}-{\rm{COOH}}\\ \,\,\,{\rm{BA}}\,{\mathrm{TM}@\mathrm{IRO}}_{{\rm{2}}}\,{\rm{NPs}}\,\,{\rm{p}} \mbox{-} \mathrm{HBA}\end{array}$$

when the ·OH radical formed on the surface of NPs reacts with benzoic acid (BA) as shown in Eq. (2), *p*-hydroxy benzoic acid (*p*-HBA) is formed. By comparing the amount of *p*-HBA formed with time, the above-mentioned influence of the ·OH radicals on the catalytic reaction can be comparatively analyzed^48,49^.

**Figure 5:**
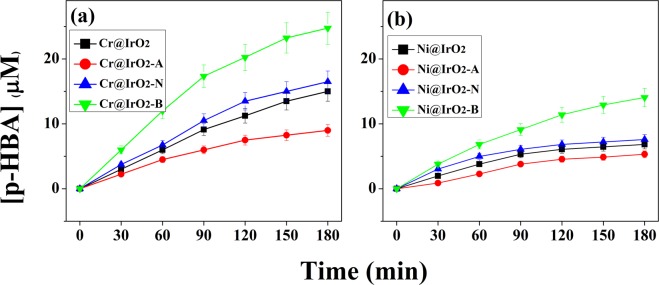
Radical formation of *p*-HBA from BA reacted with (**a**) Cr@IrO_2_ NPs and (**b**) Ni@IrO_2_ NPs. [TM@IrO_2_] = 0.4 g/L, [Benzoic acid]_0_ = 20 µM.

As can be seen in Fig. 5, the radical formation reaction of NPs synthesized under different pH conditions shows a large difference. As expected, TM@IrO_2_ NPs (a; Cr@IrO_2_ and b; Ni@IrO_2_ NPs) synthesized in the basic conditions were the most reactive regardless of the doping metal. In particular, Cr@IrO_2_-B NPs exhibited remarkably good reactivity. The results are also consistent with the results of the OER electrochemical experiment and the MB degradation experiment. In addition, it was confirmed that the tendency was similar to the change in the surface area, confirmed by the BET experiment, which provides a direct proof that the concentration of ·OH radicals existed on the surface of TM@IrO_2_ NPs are proportional to that of the defect structures.

Therefore, our results proved that the concentration of defect structures (the formation of ·OH radical) is the most important factor that influences the quality of the catalyst, which can be enhanced by the pH treatment under basic conditions. Specifically, maximizing the number of defect structures while maintaining the characteristics of IrO_2_ NPs could be the most effective way to improve the efficiency of the catalyst.

## Conclusion

Our experimental analysis showed a clear effect of pH treatment on the catalytic properties of IrO_2_ NPs. We found that Cr@IrO_2_ and Ni@IrO_2_ NPs fabricated under basic conditions, i.e., Cr@IrO_2_-B and Ni@IrO_2_-B NPs, to be the best catalysts among those tested. We specifically found that varying pH resulted in changes in the surface structure, which in turn was considered to be responsible for the changes in the catalytic properties. High-performance OER catalysts were achieved by doping the transition metals (TM = Cr or Ni) into IrO_2_ NPs, with Cr@IrO_2_-B, and Ni@IrO_2_-B NPs exhibiting the relatively small overpotential values of 258 mV and 305 mV, respectively, while other tested NPs showed poorer activities. In particular, the relatively high overpotential value and poor MB degradation of Ni@IrO_2_ NPs could be overcome by treating them with base (pH = 13.5); this pH change was found to affect the concentration of defect structures. In addition, we also found that the catalytic activity of TM@IrO_2_-B NPs is better than those of other samples by evaluating their MB degradation performances (in detail, Cr@IrO_2_-B; 35% and Ni@IrO_2_-B NPs; 55% in 180 minutes.). We also provided a direct evidence with surface morphological change and the variations of ·OH radical formations on the surface of eight tested TM@IrO_2_ NPs when the concentration of the defect structures is increased. In conclusion, we found it necessary to modulate the pH levels of the NPs during their fabrication in order to endow them with high catalytic activities.

## Methods

### Preparation of IrO_2_, Cr@IrO_2_, and Ni@IrO_2_ NP precursor solutions

Aqueous solutions of IrO_2_ NPs were prepared from potassium hexachloroiridate (K_2_IrCl_6_) and sodium hydroxide (NaOH) by following a modified version of Wohler’s method^50,51^. Upon heating, the initially dark green solution became increasingly transparent, and then turned dark blue at 90 °C, as desired. An increasing quantity of particles was seen over time after the heating was completed. After being stored at room temperature, the upper layers of the heated solution separated from the lower level of the upper stream, and a sediment eventually formed over this separation. No such sediment was found when the solution was heated to 90 °C. After preparing 1 mole % doped Cr@IrO_2_ and Ni@IrO_2_ solutions (from the dopant precursors, Cr(NO_3_)_2_∙*x*H_2_O and Ni(NO_3_)_2_∙6H_2_O, respectively), we transferred the solutions into a Teflon-lined autoclave, which was then placed for 7 h in a convection oven preheated to 220 °C, to result in the corresponding TM@IrO_2_ NP (TM = Cr or Ni) solutions, which had a gel-like appearance. We changed the pH levels of some of the samples of these solutions with HNO_3_ (acid) and of others with KOH (base), and maintained them at the target pH for 6 h. After approximately 20 min, the synthetic gel solutions became transparent. These transparent solutions were transferred into the Teflon-lined autoclaves that were then sealed and heated to 220 °C for 7 h in a convection oven. Resulting TM@IrO_2_ NPs (those subjected to acid, neutral, and base treatments are denoted as TM@IrO_2_-A, TM@IrO_2_-N, and TM@IrO_2_-B, respectively) were filtered and washed with double-distilled water (DDW) to remove any residue. Using this procedure, we prepared eight different IrO_2_ NP-containing samples.

### Characterization

Surface morphologies of the prepared samples were measured by using a field-emission scanning electron microscope (FE-SEM, FEI Inspect F50) operating at 15 kV. Surface area analyses were performed by the Brunauer-Emmett-Teller (BET) method using an Autosorb-iQ 2ST/MP apparatus (Quanta chrome). Electronic structures of the samples were analyzed using the high-resolution photoemission spectroscopy (HRPES) at the 8A1 beamline (the Pohang Accelerator Laboratory)^29^. Methylene blue (MB: 0.15 mM) degradation of Cr@IrO_2_ and Ni@IrO_2_ NPs was measured at regular intervals, mixed immediately with 1 mL of methanol as inhibitor, and then filtered. Real-time concentration variation was measured using an UV–Vis spectrophotometer (UV–2600, SHIMADZU) at 484 nm. Electrocatalytic activities of prepared Cr@IrO_2_ and Ni@IrO_2_ NPs were compared by carrying out the OER in 1 M KOH electrolyte. Each of the eight samples (10 mg) was dispersed in a solution containing either Nafion (100 μL; 5wt %, DuPont) or absolute ethanol (2 mL; Daejung, 99.9%) by ultra-sonicating the mixture for 30 min. The electrochemical OER activities were then measured in 1 M KOH (Aldrich, 90%) by using a potentiostat (Ivium Technologies; scan rate of 10 mV/sec). The results of the repeated measurements for each sample varied by ± 4.5%. The radical formation of benzoic acid (20 µM) for the eight tested samples (0.4 g/L) was measured using a 300-W Xe arc lamp (NEWPORT 300 W Xenon Light Source) with a 320 nm cut-off filter.
